# Carbon Nanotube-Based Biosensors for Non-Invasive Biofluid Analysis

**DOI:** 10.3390/bios16080431

**Published:** 2026-08-07

**Authors:** Samriddha Dutta, Ashok Mulchandani

**Affiliations:** Department of Chemical & Environmental Engineering, University of California, Riverside, Riverside, CA 92521, USA; sdutt013@ucr.edu

**Keywords:** carbon nanotube, biosensor, non-invasive biofluid, biomarker

## Abstract

Carbon nanotube (CNT)-based biosensors have emerged as promising platforms for non-invasive biofluid analysis because of their high electrical conductivity, large surface area, tunable optical properties, and versatile surface chemistry, enabling miniaturized, flexible sensing devices. Sweat, saliva, tears, and urine are increasingly recognized as attractive alternatives to blood for point-of-care diagnostics because they enable repeated, non-invasive sampling while containing clinically relevant metabolites, electrolytes, proteins, hormones, nucleic acids, pathogens, and other biomarkers. However, the low abundance of many analytes, matrix complexity, biofouling, and biofluid-specific variability present significant analytical challenges. This review critically examines the different CNT-based sensor architectures, and their recent advances in non-invasive analysis of sweat, saliva, tears, and urine. It integrates sensor architecture, biofluid-specific analytical challenges, sample-validation level, and translational readiness within a single comparative framework. Representative applications are discussed for metabolic monitoring, renal health assessment, infectious disease testing, and other clinically relevant uses. Beyond clinical diagnostics, emerging non-clinical applications, including drug-of-abuse detection, forensic body-fluid identification, and occupational or environmental exposure assessment, are also highlighted. Finally, we discuss key barriers limiting real-world translation of CNT biosensors, including material reproducibility issues, biofouling, physiological interpretation of biofluid biomarkers, scalable manufacturing, and long-term operational stability, and outline future strategies to advance these platforms toward robust, reliable, and widely deployable biosensing technologies.

## 1. Introduction

Non-invasive biofluid analysis has become a major focus in modern diagnostics. By utilizing easily accessible matrices like sweat, saliva, tears, and urine, it provides critical physiological insights without the pain, infection risks, or practical challenges associated with invasive sampling methods such as venipuncture [1,2,3,4,5,6,7,8,9]. As healthcare increasingly shifts toward portable systems, these repeatable, user-friendly sampling methods have become ideal for point-of-care (POC) testing, wearable monitoring, and home-based diagnostics [1,2,3,4,5,6,7,8,9]. These biofluids contain a diverse array of analytes, such as metabolites, electrolytes, proteins, hormones, nucleic acids, pathogens, and drugs, thereby supporting a broad spectrum of applications, ranging from health monitoring to infectious disease testing, forensic analysis, and environmental exposure assessments [1,2,4,5,6,7,10,11,12,13].

Detection and quantification of these analytes, particularly in decentralized formats, typically rely on biosensors. A biosensor is a device that measures biological or chemical reactions by generating signals proportional to the concentration of an analyte in the reaction [14]. A typical biosensor comprises a bioreceptor, a transducer, signal-processing electronics, and an output interface. The bioreceptor, which may be an enzyme, antibody, aptamer, nucleic acid, or whole cell, selectively interacts with the analyte and produces a physicochemical change, such as a variation in charge, mass, pH, heat, or optical properties. The transducer converts this biorecognition event into a measurable electrical or optical signal related to the analyte concentration. The signal is subsequently conditioned, amplified, and digitized by the electronic circuitry before being presented in a numerical, graphical, tabular, or image-based format suitable for interpretation by the end user [14].

Despite their advantages, non-invasive biofluids present several analytical challenges. Biomarker concentrations are often substantially lower than those found in blood and can vary widely depending on factors such as collection method, hydration status, secretion rate, circadian rhythm, and individual physiology [1,3,5,15,16,17]. Matrix effects, contamination, and biofluid-specific variability further complicate the challenges of biomarker quantification and interpretation [1,3,15,18]. As a result, biosensors developed for non-invasive biofluid analysis need to demonstrate robustness, selectivity, and sensitivity with high reliability across complex environments.

Recent advances in nanomaterials, flexible electronics, and wireless systems have significantly accelerated the development of non-invasive biosensing platforms [3,19,20]. For example, wearable sensors have already been reported for monitoring sweat metabolites, electrolytes, hormones, and nutritional markers on the body [3,19,20,21]. It is worth mentioning, however, that the majority of these platforms are still proof-of-concept demonstrations in buffer, artificial matrix, or small pilot studies.

Among the nanomaterials explored for biosensing applications, carbon nanotubes (CNTs) have emerged as one of the most promising candidates for transducer material because they combine high aspect ratio, large effective surface area, excellent electrical conductivity, tunable electronic and optical properties, and strong sensitivity to local chemical and biological perturbations, making them highly responsive elements for biosensor design [22,23,24,25,26]. Their surface chemistry further supports sensor development as their graphitic sidewalls can be covalently or non-covalently functionalized with enzymes, antibodies, aptamers, or nucleic acids, enabling tailored biorecognition interfaces that allow both label-free and labeled detection [22,23,24,25,26]. CNTs can also be prepared as inks, films, or hybrid composites making them suitable for integration into flexible, paper-based, textile, and wearable formats [20,21,22,27]. These characteristics make CNT-based biosensors particularly well suited to the demands of non-invasive biofluid analysis. Their compatibility with low-cost substrates and printing methods supports use in point-of-care and home-use settings.

Nevertheless, several challenges continue to hinder the widespread translation of CNT-based biosensors [23,28]. The electronic properties of CNT networks are sensitive to nanotube chirality distribution, defect density, bundling, and dispersion quality, which can introduce device-to-device variability and complicate large-scale manufacturing [28,29,30]. Long-term operational stability in complex biofluids may be limited by biofouling, degradation or desorption of biorecognition elements, and changes in CNT–electrolyte interfacial properties. Moreover, reliably linking biofluid biomarker concentrations to physiological or pathological conditions requires enhanced calibration strategies and established sampling procedures combined with contextual physiological data [1,28,31].

**Figure 1:**
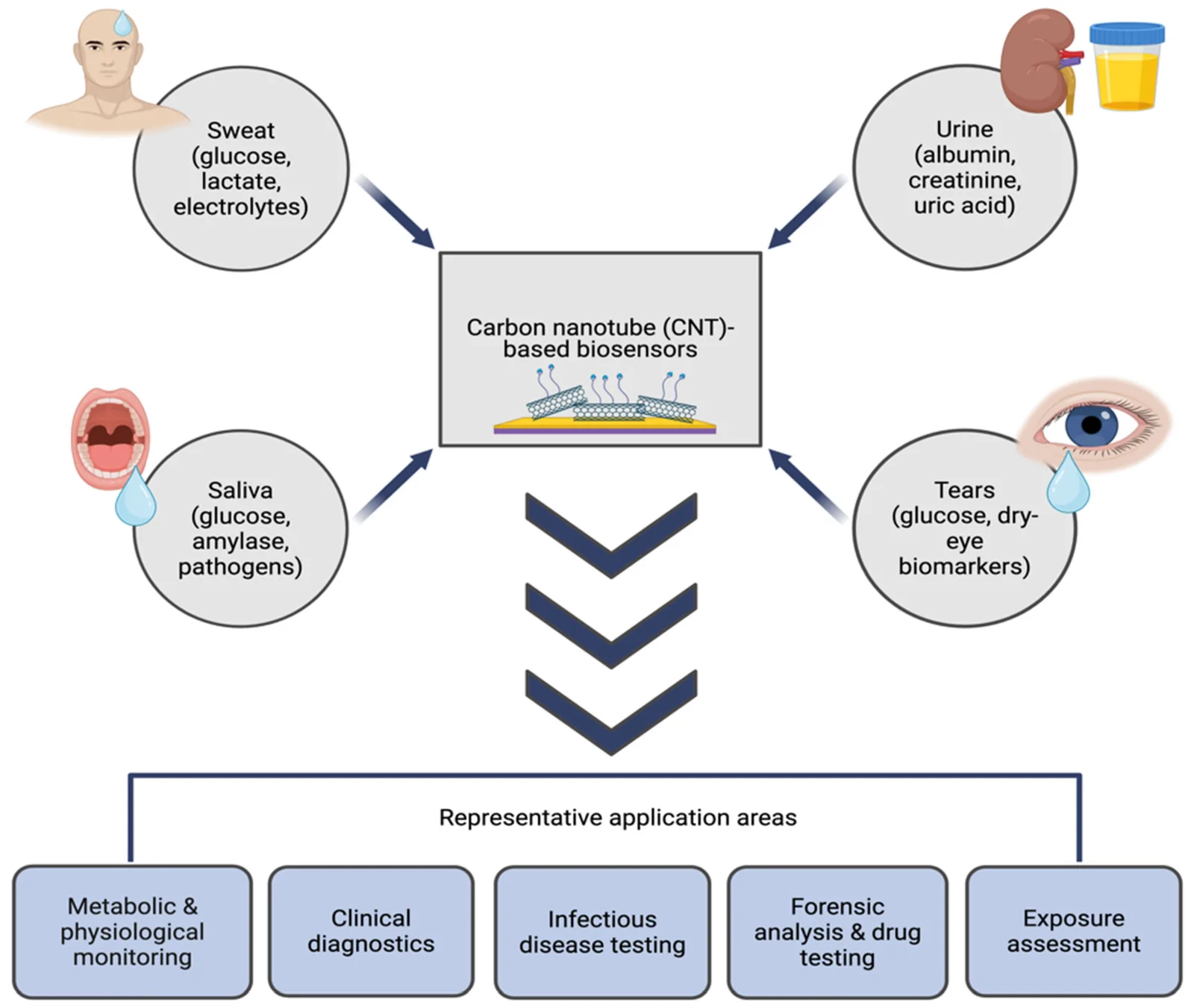
Representative landscape of CNT-based biosensors for non-invasive biofluid analysis (Created in BioRender. Dutta, S. (2026) https://BioRender.com/ejpk41s).

Given the rapid expansion of both CNT biosensor research and non-invasive biofluid analysis, there is a need for a focused review integrating these two rapidly evolving fields. While recent reviews have discussed CNT-based biosensors, wearable sensing technologies, or non-invasive biofluid diagnostics from broader or individual perspectives, a comprehensive assessment of CNT-based biosensors specifically for non-invasive biofluid analysis remains limited [1,17,28,32]. The present review integrates the major CNT sensing architectures, including field-effect transistor, chemiresistive, electrochemical, and optical platforms, with applications in sweat, saliva, tears, and urine. Particular attention is given to the sample matrix and stage of analytical evaluation, distinguishing proof-of-concept studies conducted in buffer, artificial biofluids, or spiked samples from studies evaluated using authentic human biofluids. The review also considers both clinical and emerging non-clinical applications and examines the principal barriers affecting translation, including material reproducibility, biofouling, physiological interpretation, long-term stability, and scalable manufacturing. Through this combined scope, the review aims to clarify the progress of CNT-based sensing platforms toward practical non-invasive biofluid analysis and identify the areas in which further validation is needed. Figure 1 presents the representative landscape of CNT-based non-invasive biofluid biosensors, illustrating their applications across sweat, saliva, tears, and urine, along with key analytes and major application domains.

## 2. Carbon Nanotube Fundamentals and Transduction Mechanisms

Carbon nanotubes (CNTs) are essentially quasi-one-dimensional allotropes of carbon formed by rolling graphene sheets into cylindrical structures with nanometer-scale diameters ranging from 0.4 nm to 6 nm, and lengths varying from tens or hundreds of nanometers to numerous micrometers and, in optimized growth conditions, reaching millimeter or even centimeter scales [33,34,35]. In their simplest form, single-walled carbon nanotubes (SWCNTs) consist of a single graphitic shell, whereas multi-walled carbon nanotubes (MWCNTs) contain multiple concentric single walled graphene cylinders held together by Van der Waals forces with an interlayer spacing of ~3.4 Å [35,36,37]. The arrangement of carbon atoms around the tube circumference, described by the chiral indices (n,m), determines whether an SWCNT behaves as a metallic or semiconducting conductor [23,35,37]. This intrinsic link between structure and electronic behavior, coupled with high charge-carrier mobility, large surface area and high mechanical strength, makes CNTs especially attractive as active materials in biosensors, where subtle molecular recognition events occurring near the nanotube surface must be converted into measurable signals [22,23].

In addition to the tunable electronic behavior, CNTs present excellent mechanical and thermal properties. Individual SWCNTs exhibit Young’s moduli on the order of 1 TPa and tensile strengths ranging from tens to over one hundred gigapascals, similar to, or even surpassing, high-performance engineering materials [26,38,39,40]. However, it is important to note that these values represent idealized, defect-limited isolated tubes, and bulk assemblies like films, ropes, and arrays display much lower moduli and strengths [41]. This gap between intrinsic and ensemble properties must be considered in device design. Similarly, thermal conductivities of approximately 3000–3500 W m^−1^ K^−1^ have been measured for isolated individual SWCNTs at room temperature, though this does not translate directly to CNT networks or films [40,42]. At the device level, CNT inks and dispersions can be processed into thin, percolating films that combine high conductivity with mechanical compliance and, when deposited at low coverage, appreciable optical transparency [43]. These properties are especially important for conformal, skin-mounted patches, and electrodes incorporated with textiles, among others.

**Figure 2:**
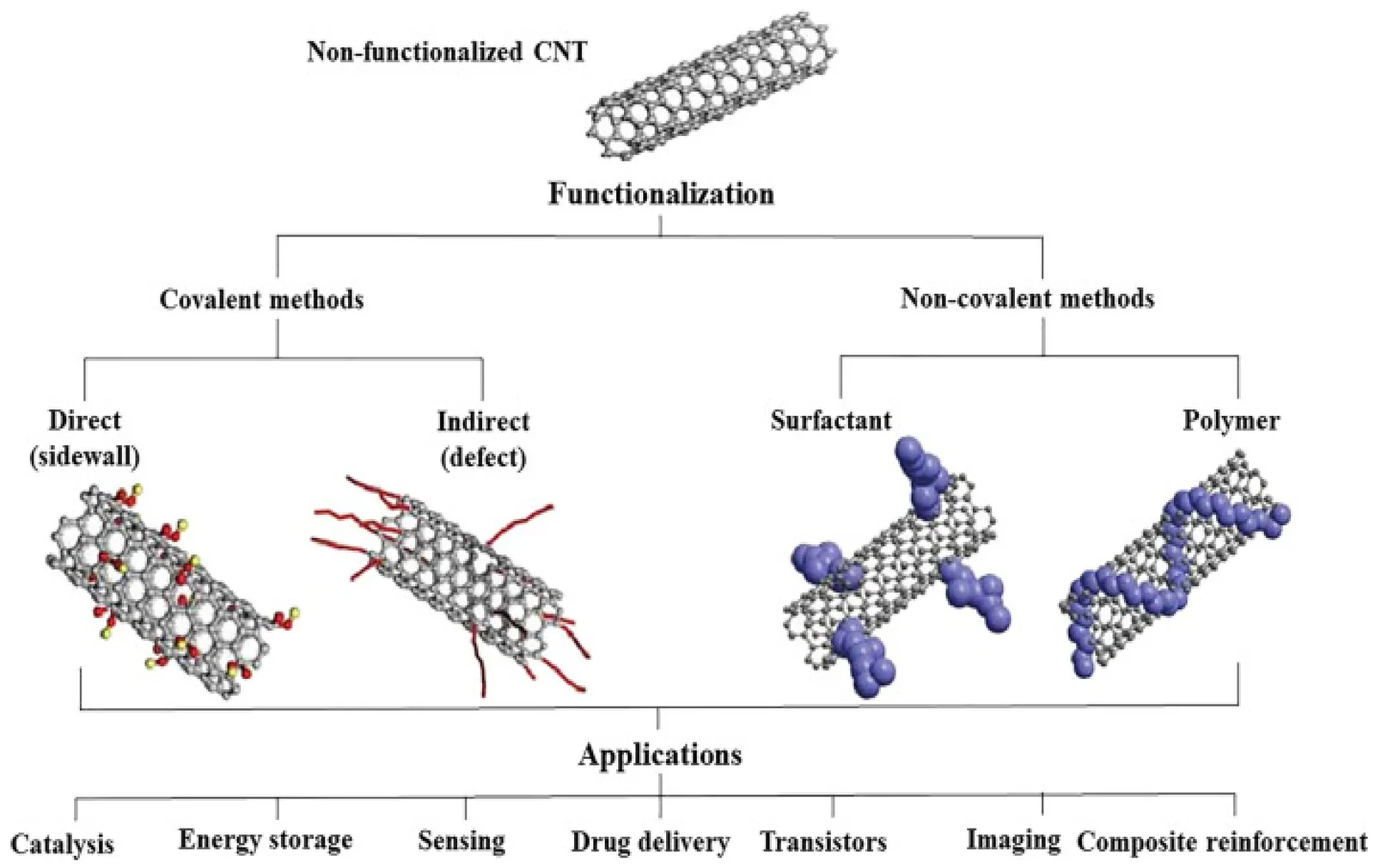
Common functionalization methods and applications of CNTs (Reproduced from ref [45] http://creativecommons.org/licenses/by/4.0/, accessed on 5 July 2026).

Surface chemistry of CNTs is equally important for biosensor design. Their surfaces can be engineered to facilitate biomolecule immobilization generally through covalent or noncovalent functionalization [37,44,45,46]. Figure 2 highlights some of the common functionalization methods and applications of CNTs. Covalent methods provide strong and stable attachment sites, but extensive sidewall modification may disrupt the sp^2^ carbon network and potentially alter the intrinsic electronic and optical properties of the nanotubes [45]. In contrast, noncovalent strategies preserve the underlying π-conjugated framework of CNTs, while also improving dispersibility and providing additional functional handles [46]. Because these interactions are generally weaker, the long-term stability of the functional layer may depend on environmental and processing conditions, such as pH and temperature, which can promote molecular desorption or surface rearrangement [47]. Together, these approaches enable the integration of a broad range of biorecognition elements, including enzymes, antibodies, aptamers, nucleic acids, ionophores, and molecularly imprinted polymers, to the CNT network. Beyond classical covalent and noncovalent functionalization, mechanically interlocked derivatives of SWCNTs (MINTs) represent an emerging functionalization strategy that combines the robust retention associated with covalent modification with preservation of the native sp^2^–carbon lattice. In MINTs, macrocycles are mechanically trapped around the nanotubes rather than directly bonded to or simply adsorbed onto their surfaces, providing a stable yet structurally non-disruptive platform for introducing additional functionality [48,49,50,51]. Recent work has extended MINT synthesis to aqueous environments, suggesting potential relevance for future biosensing interfaces intended to operate in aqueous media [51].

Collectively, these electronic, mechanical, and chemical attributes have enabled the development of a diverse range of CNT-based biosensor architectures. CNTs have been employed as the conductive channel in field-effect transistor (FET) biosensors, where binding-induced changes in local electrostatics modulate channel conductance; as percolating networks in chemiresistive devices, where adsorption or charge-transfer events alter network resistance; as high-surface-area modifiers on electrochemical electrodes, where they enhance electron-transfer kinetics and catalytic currents; and as near-infrared fluorescent emitters in optical nanosensors based on semiconducting SWCNTs [23,26,28,52,53]. Each of these transduction modes offers its own balance of sensitivity, stability, form factor, and fabrication complexity. Understanding these mechanisms is essential for evaluating how CNT-based biosensors can be tailored for the detection of biomarkers in fluids.

**Figure 3:**
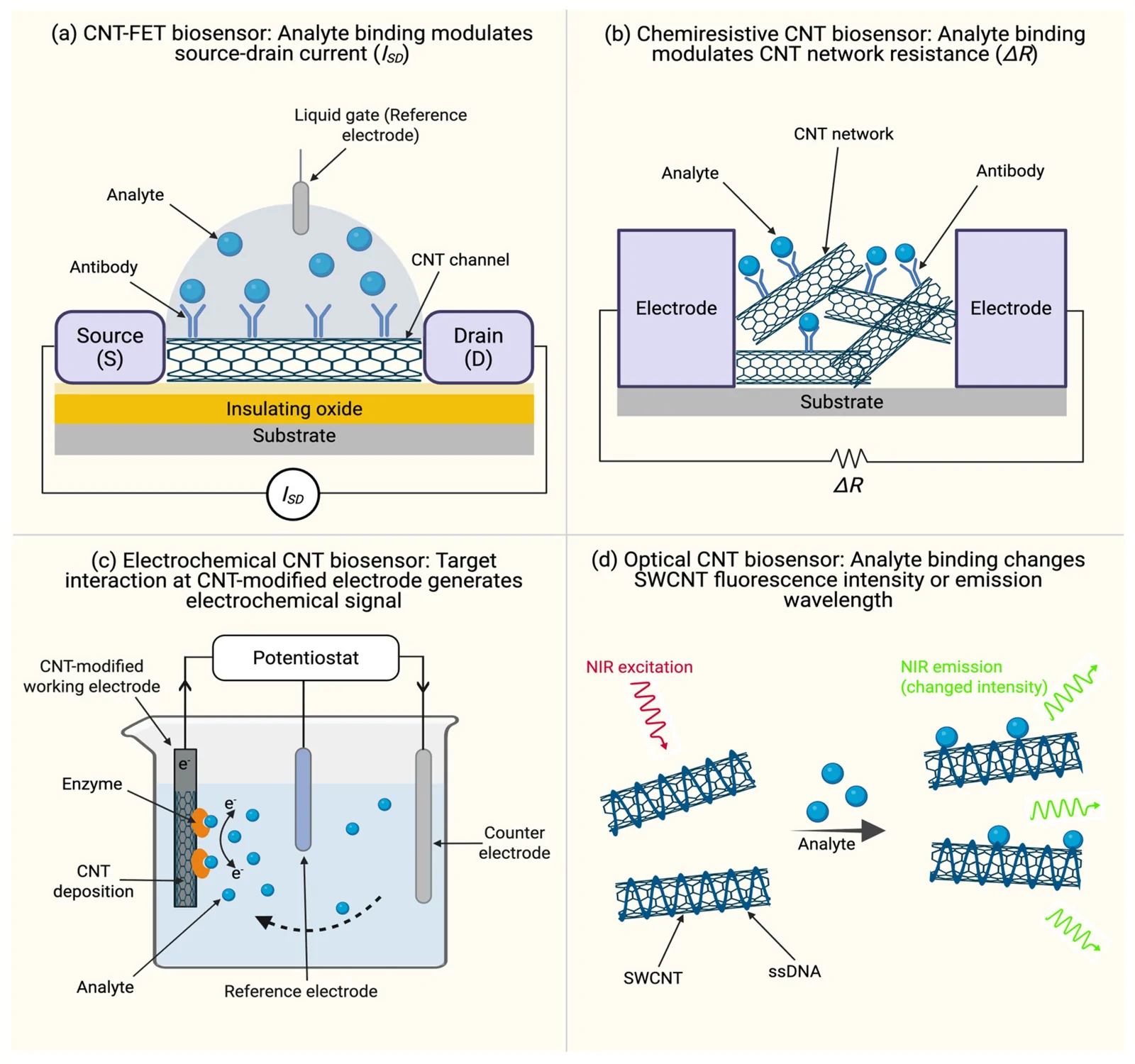
Schematic representation of the principal transduction mechanisms of CNT-based biosensors. (**a**) In a CNT field-effect transistor biosensor, analyte binding to CNT-immobilized antibodies modulates the source–drain current. (**b**) In a chemiresistive CNT biosensor, analyte binding to antibodies immobilized on the CNT network changes its electrical resistance. (**c**) In an electrochemical CNT biosensor, enzymatic interaction with the analyte at a CNT-modified working electrode generates a measurable electrochemical signal. (**d**) In an optical biosensor, ssDNA noncovalently wraps around the SWCNT to form an analyte-responsive corona, and analyte interaction changes the NIR fluorescence intensity or emission wavelength. (Created in BioRender. Dutta, S. (2026) https://BioRender.com/ejpk41s).

This section summarizes the operating principles and analytical performance of these architectures using representative studies from a range of sample matrices. Their applications specifically in sweat, saliva, tears, and urine are discussed in the subsequent sections. Figure 3 provides schematic representations of the different sensor architectures, while Table 1 summarizes the role of CNTs in each architecture, the corresponding signal-generation mechanisms, and their principal strengths and limitations.

### 2.1. CNT-Based Field-Effect Transistor Biosensors

In CNT-FET biosensors, one or more semiconducting CNTs span source and drain electrodes, forming the conducting channel. A small source–drain voltage ($V_{DS}$) drives charge carriers through the CNT, generating a measurable drain current ($I_{D}$). A third electrode, the gate, applies an electric field that regulates the carrier density within the CNT channel and thereby controls its conductivity; changing the gate voltage ($V_{G}$) changes $I_{D}$, which is the basic principle underlying transistor operation [30,54,55]. In liquid-gated CNT-FETs, the gate is a reference electrode immersed directly in the sample solution rather than separated by a solid dielectric. The gate potential is transmitted to the CNT channel through the electrolyte via electric double layers that form at both the gate–electrolyte and CNT–electrolyte interfaces; because these double layers behave as very high-capacitance interfaces, they allow efficient channel modulation even at low applied voltages [30,55].

To facilitate biosensing, the CNT surface is functionalized with biorecognition elements such as antibodies, aptamers, enzymes, or DNA probes. When a target biomolecule binds to the functionalized surface, it alters the local electrical environment around the nanotube. If this binding occurs within the effective Debye screening length ($\lambda_{D}$), the resulting electrostatic perturbation modulates the CNT’s carrier density and produces a measurable shift in $I_{D}$ [56]. Depending on device architecture, binding may also contribute to the signal through direct charge transfer or by modulating the Schottky barriers at the metal–CNT contacts. Because CNTs have a one-dimensional structure with a high surface-to-volume ratio, even small electrostatic perturbations near the surface produce large relative changes in channel conductance, enabling CNT-FET biosensors to achieve rapid, label-free, and highly sensitive biomolecular detection [23,28,54,57].

CNT-FET biosensors have obtained femtomolar and femtogram per milliliter range detection limits for analytes including proteins, nucleic acids, viral antigens in buffer and serum; for example, He et al. reported an aptamer-modified CNT-FET for continuous glucose monitoring with a limit of detection of 0.5 fM [58]. In another study by Li et al., exosome-related target Mucin1 (MUC1) was detected at 0.34 fg mL^−1^ using functionalized CNT-FET biosensors [59]. CNT-FET biosensors have also been used to detect SARS-CoV-2 antigens in buffer and clinical nasopharyngeal swab specimens, highlighting their utility for viral diagnostics [60,61].

These findings highlight the strong potential of CNT-FET biosensors for detecting low-abundance biomarkers relevant to non-invasive biofluids. Their high surface-to-volume ratio and label-free electrical readout make them well suited for sensitive, real-time biomolecular detection. However, CNT-FET performance remains strongly influenced by CNT purity, metallic/semiconducting composition, network density, and device-to-device variability. Furthermore, in complex biofluids, additional challenges such as Debye screening, nonspecific adsorption, and biofouling can reduce sensitivity and long-term stability [30,54]. Therefore, although CNT-FETs are powerful platforms for highly sensitive chip-based sensing, their broader translation will require improved CNT material uniformity, robust antifouling interfaces, optimized receptor design, and scalable fabrication strategies.

### 2.2. Chemiresistive CNT Biosensors

Chemiresistive CNT biosensors use a CNT film or network as a conductive channel between two electrodes whose conductance or resistance changes when analytes interact with its surface. In such systems, a constant voltage or current is applied across the CNT percolation network, and biorecognition elements, typically antibodies, aptamers, or enzymes, are immobilized on the CNTs using pyrene linkers, polymers, or covalent coupling. Analyte binding events modulate charge density, carrier scattering, and inter-tube junction characteristics, producing measurable resistance changes that provide information on target presence and concentration. This two-terminal architecture maintains structural simplicity while providing biochemical selectivity, making chemiresistive CNT biosensors attractive for low-cost, printed, and paper-based formats [23,26,52].

Chemiresistive CNT biosensors have been developed for a range of analytes including proteins, metabolites, microorganisms and small molecules, often achieving nanomolar to picomolar detection limits under controlled conditions [52,62,63,64,65]. For instance, Cella et al. reported a highly sensitive SWCNT chemiresistive affinity sensor for the detection of uncharged or weakly charged small molecules [63]. The sensor detected glucose via the displacement of plant lectin, Concavalin A, bound to polysaccharide, dextran, immobilized on SWCNTs, and exhibited picomolar sensitivity to glucose and high selectivity over human serum proteins and other sugars. In another study, Puri and co-workers described a label-free SWCNT chemiresistive biosensor for the cardiac biomarker myoglobin, with a linear conductance response over 1.0–1000 ng mL^−1^ and an approximate sensitivity of 118% per decade [64]. Prakash et al. also described an ultrasensitive chemiresistive biosensor for label-free detection of myoglobin where MWCNTs embedded in SU-8 nanofibers, functionalized with myoglobin antibodies, achieved a limit of detection of 6 fg mL^−1^ [65]. Paper-based chemiresistive CNT devices have also been realized; for example, Shen and group reported an SWCNT chemiresistive biosensor on paper that enabled quantitative and selective detection of human serum albumin with a limit of detection of 1 pM [52].

In the context of non-invasive biofluids, these features are particularly appealing because they allow detection of low concentrations of biomarkers using simple, flexible and disposable substrates. However, chemiresistive responses are also susceptible to environmental and matrix-related influences including humidity, nonspecific adsorption. Small variations in CNT network can also generate substantial device-to-device differences [66,67]. So careful control of CNT synthesis, deposition protocols, and surface functionalization, and, where feasible, on-chip reference or control channels are therefore essential for distinguishing true analyte responses from nonspecific effects.

### 2.3. Electrochemical CNT Biosensors

Electrochemical biosensors are among the oldest categories of biosensors, and to date they are highly popular because of their relative simplicity, low cost, and compatibility with portable instrumentation [23]. Typical CNT-based electrochemical biosensors contain a CNT-modified working electrode together with reference and counter electrodes, although two-electrode and integrated printed configurations are also widely used. The working electrode is functionalized with biological recognition elements such as enzymes, antibodies, or nucleic acids, and target recognition or an associated biochemical reaction is transduced into measurable electrical signals, including changes in current, potential, or impedance, that are correlated with analyte concentration. CNTs act as electrode modifiers that increase the electroactive surface area, enhance electron transfer kinetics, and provide robust scaffolds for biomolecule immobilization, thereby improving sensitivity and lowering detection limits [23,36].

Because they build upon well-established electrochemical techniques, CNT-modified electrodes have been widely explored for the detection of diverse analytes, including metabolites, proteins, nucleic acids, and hormones in buffer solutions and conventional biological matrices such as blood, serum, and plasma, and also in non-invasive biofluids, particularly saliva, sweat, and urine. For example, Claussen et al. developed a glucose oxidase-functionalized CNT electrode decorated with platinum nanospheres that achieved a detection limit of 380 nM and a linear response over physiologically relevant glucose concentrations in phosphate-buffered saline [68]. CNT-enhanced electrochemical aptasensors have also been developed for protein biomarkers such as myoglobin, with Zhu et al. demonstrating successful detection with picogram-per-milliliter sensitivity in human serum using a CNT-gold nanoparticle based self-powered biosensor [69]. Similarly, Tian et al. reported a MWCNT–gold nanoparticle electrochemical immunosensor for prostate-specific antigen (PSA) with a detection limit of 7 pg mL^−1^ in human serum [70]. Electrochemical CNT platforms have also been widely applied to nucleic acid sensing, with numerous DNA and microRNA biosensors reporting picomolar to femtomolar detection limits through hybridization-based electrochemical assays [36,71,72].

Collectively, these studies highlight the enhanced sensitivity conferred by CNT-modified electrodes and their potential for detecting the low concentrations of biomarkers encountered in non-invasive biofluids. However, these biosensors face some major challenges which include electrode fouling in protein-rich biofluids, potential drift when reference and counter electrodes are miniaturized, and the need to package all electrodes into compact, mechanically robust devices [73,74]. Nonetheless, the relative maturity of electrochemical platforms and their compatibility with existing point-of-care infrastructure render these CNT-based electrochemical biosensors among best options for CNT-enabled diagnostics, including applications in non-invasive biofluid analysis.

### 2.4. Optical CNT Biosensors

Optical CNT biosensors use the optical properties of semiconducting SWCNTs, most commonly their intrinsic near-infrared (NIR) fluorescence, to detect target molecules, although Raman scattering and optical absorption have also been used for sensing [23,24]. To provide molecular specificity, SWCNTs are functionalized or interfaced with polymers, DNA, aptamers, antibodies, or other recognition layers. When a target analyte binds to or interacts with a recognition layer at the nanotube surface, it changes the local chemical environment of the SWCNT, producing measurable changes in fluorescence intensity, emission wavelength, or fluorescence lifetime. Because SWCNTs emit in the NIR region where biological samples show relatively low background fluorescence, in vivo applications can benefit from this. Moreover, the SWCNT fluorescence does not show blinking or bleaching [24,75].

A major advancement in the field was the introduction of corona phase molecular recognition (CoPhMoRe) by Zhang and co-workers, in which synthetic polymers adsorbed on SWCNTs create selective recognition sites while the nanotube acts as the optical transducer [76]. Earlier, Barone et al. reported NIR optical SWCNT sensors using beta-D-glucose as a model analyte, helping establish the basis for optical CNT biosensing [77]. Since then, optical SWCNT nanosensors have been developed for a range of analytes including neurotransmitters, proteins, lipids, reactive oxygen species, hormones, and small-molecule metabolites. For example, Kruss and co-workers used CoPhMoRe to identify dopamine-responsive DNA and RNA corona phases on fluorescent SWCNTs, achieving reversible dopamine detection in solution with an 11 nM limit of detection [78]. Bisker et al. extended CoPhMoRe to protein sensing, demonstrating selective fibrinogen detection in human serum with >80% fluorescence quenching at saturation [79] Bisker and co-workers also reported CoPhMoRe-based insulin detection and later applied SWCNT optical nanosensors to monitor insulin secretion from pancreatic β cells [80,81].

However, most optical CNT biosensors have only been demonstrated in buffered solutions, serum, or other controlled laboratory conditions. Translation to non-invasive biofluids will require maintaining stable optical signals in complex biological matrices, developing antifouling recognition layers that retain high selectivity, and improving the reproducibility and biocompatibility of CNT functionalization [75,82,83]. Consequently, optical CNT biosensors remain highly promising, but still relatively early-stage sensing platforms for non-invasive biofluid analysis.

**Table 1:** Comparison of major CNT-based biosensor architectures and transduction mechanisms.

Mode	Typical CNT Role	Main Signal Mechanism	Main Strengths	Main Limitations
**CNT-FET**	Semiconducting CNTs form the conducting channel between source and drain electrodes.	Target recognition near the CNT surface changes the local electrical environment and modulates the drain current.	High sensitivity; rapid, label-free detection; compact and low-voltage operation.	Device variability; Debye screening; nonspecific adsorption and biofouling.
**Chemiresistive**	A CNT film or network forms the conductive pathway between two electrodes.	Analyte interaction alters charge transport through the CNT network, producing a change in resistance or conductance.	Simple two-terminal design; low-cost readout; compatible with printed, flexible, and paper-based devices.	Environmental and matrix effects; nonspecific adsorption; variability in CNT network properties.
**Electrochemical**	CNTs modify the working electrode, increase electroactive surface area, and support bioreceptor immobilization.	Recognition or biochemical reactions generate changes in current, potential, or impedance; CNTs increase electroactive area and often improve electron-transfer kinetics.	Established portable readout; broad analyte compatibility; suitable for printed and point-of-care devices.	Electrode fouling; electroactive interferents; device-integration challenges.
**Optical**	Semiconducting SWCNTs serve as optical transducers, most commonly through intrinsic NIR fluorescence.	Target interaction at the functionalized SWCNT surface changes fluorescence intensity, emission wavelength, or lifetime; Raman or absorption changes may also be used.	High photostability; relatively low biological background in the NIR region; potential for remote and multiplexed sensing.	Matrix interference; recognition-layer instability and fouling; specialized instrumentation; limited validation in authentic biofluids.

## 3. CNT-Based Non-Invasive Biofluid Biosensors for Health Assessment and Disease Monitoring

Having outlined the basic CNT sensing architectures, advantages, and key considerations for non-invasive biofluid analysis, this section focuses on the health-monitoring and disease-related applications of CNT-based biosensors across different non-invasive biofluids. Sweat, saliva, tears, and urine provide insight into various aspects of human physiology and disease, but each presents distinct analytical challenges. The following subsections highlight representative CNT-based biosensors, with emphasis on target biomarkers, sensing strategies, analytical performance, and translational relevance. The studies discussed in this section represent different stages of validation, ranging from proof-of-concept testing in buffer, artificial biofluids, and spiked samples to evaluation using authentic human samples and clinical specimens. The sample matrix and stage of validation are therefore specified for each study to distinguish preliminary analytical demonstrations from clinically evaluated platforms. Table 2 summarizes the representative biosensors, including the target analyte, CNT material, sensing method, sample type, analytical range, limit of detection, and extent of testing in real human samples, thereby providing a comparative indication of their translational readiness.

### 3.1. Sweat

Sweat is one of the most developed non-invasive biofluids for wearable CNT-based biosensors because it is readily available at the skin surface and can be sampled repeatedly without needles or invasive procedures [1,17]. This has made sweat an attractive matrix for flexible sensing platforms, including skin-mounted patches, textile-integrated devices, and glove-based sensors [17,84]. Wearable sweat sensors also support real-time and continuous monitoring, which is useful for tracking dynamic physiological changes during exercise, stress, or daily activity. Most CNT-based sweat biosensors reported to date use electrochemical sensing. The most commonly targeted sweat analytes include metabolites such as glucose, lactate, and uric acid, as well as electrolytes such as Na^+^, K^+^, Cl^−^, and pH. These markers are relevant to metabolic activity, exercise physiology, hydration status, electrolyte balance, and even clinical testing [17].

Glucose has been a major target for wearable sweat biosensors because of its relevance to diabetes monitoring. However, sweat glucose occurs at much lower levels than blood glucose, which is normally around 3.9–5.5 mM in healthy individuals [85]. In comparison, sweat glucose is typically reported at 0.06–0.11 mM in healthy people and 0.11–1 mM in diabetic individuals; so, highly sensitive sensors are required [86]. Sweat levels can also be affected by sweat rate, glandular transport, local metabolism, skin contamination, and temporal lag relative to blood glucose which remains a challenge [87]. Shamili et al. reported an all-printed flexible electrochemical biosensor to quantify the amount of glucose in human sweat [84]. The working electrode of the sensor was printed using a customized ink containing multi-walled carbon nanotube (MWCNT), poly (3,4-ethylenedioxythiophene) polystyrene sulfonate (PEDOT: PSS), and iron (II, III) oxide (Fe_3_O_4_) nanoparticles. The sensor achieved a linear range of 1–400 μM and a detection limit of approximately 0.38 μM in buffer, and a linear range of 10–200 μM and a detection limit of approximately 0.31 μM in artificial sweat. Results from real-time on-body monitoring during physical activity also correlated well with a commercial blood glucose meter. In another study by Zheng et al., the authors developed a wearable enzymatic sweat glucose biosensor based on a porous polyglycolic acid-carbon nanotube (PGA-CNT) electrode loaded with glucose oxidase (GOx). The 3D PGA-CNT network provided a high-specific-surface-area scaffold for stable GOx immobilization and improved charge transfer, enabling flexible and stable electrochemical detection under bending. The biosensor showed a linear glucose response from 20–300 μM with a detection limit of 5.18 μM in buffer, demonstrating the potential of porous CNT-based electrode architectures for sweat glucose monitoring. The sensor showed excellent selectivity against common interferents (lactic acid, uric acid, ascorbic acid) and it was also validated in artificial sweat with high accuracy [88].

**Figure 4:**
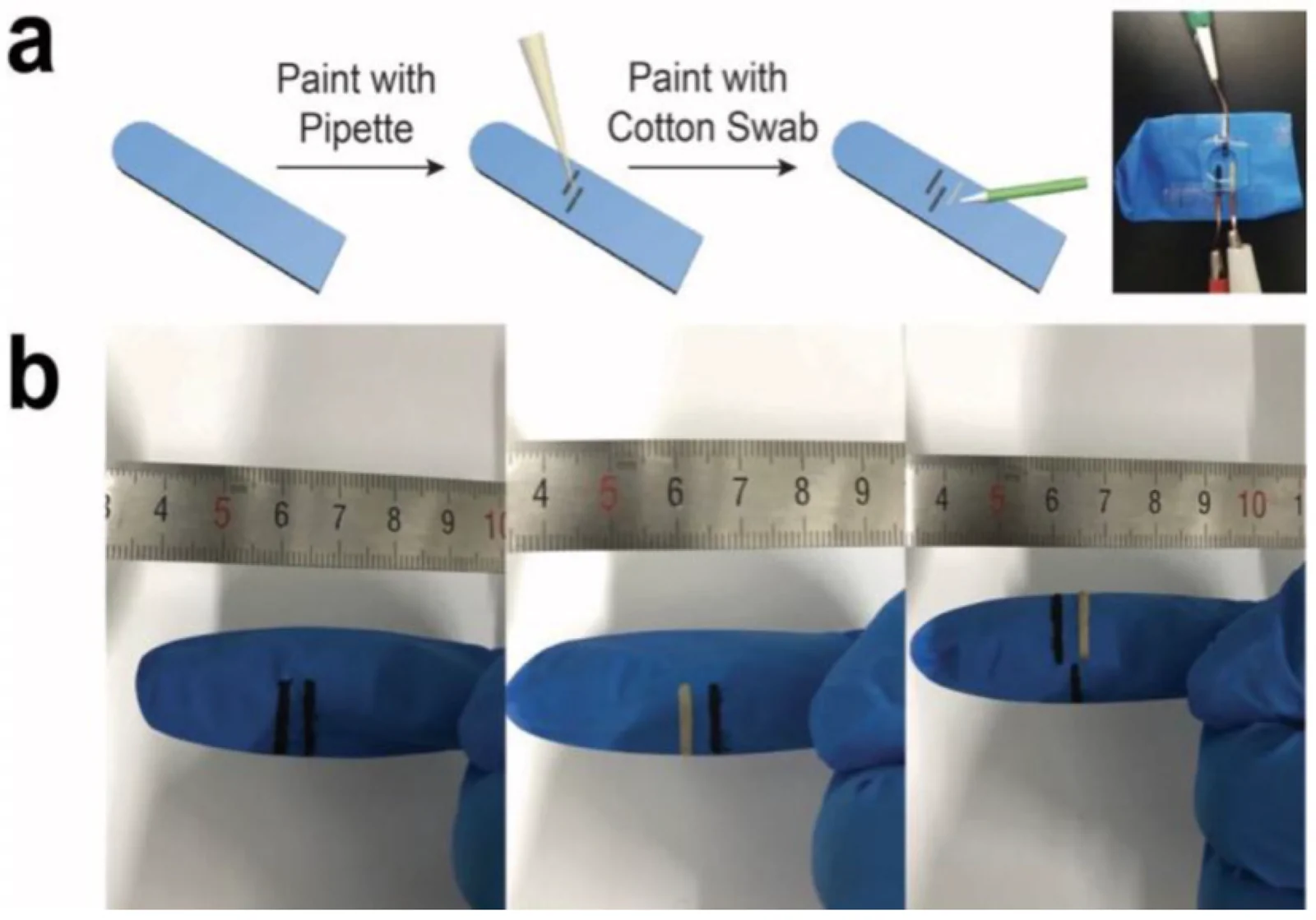
Schematic of the fabrication process of the CNT-based amperometric lactate biosensor and the connection method. (**a**) From left to right: Blank glove; Depositing CNT solution onto the glove surface with auto pipettes; painting the Ag/AgCl electrode with cotton swabs; connecting electrodes with potentiostat with copper wires. (**b**) Camera images of the CNT-based glove biosensors. Left: a sensor based on two CNT electrodes. Middle: a sensor based on a CNT and an Ag/AgCl electrode. Right: a sensor based on two CNT electrodes and an Ag/AgCl electrode. (Adapted from ref. [89]).

Lactate is another studied sweat metabolite because it reflects muscle activity and exercise physiology. For example, Luo et al. developed a wearable CNT-based amperometric lactate biosensor fabricated directly on glove fingers by painting CNT and/or Ag/AgCl inks onto the flexible substrate and immobilizing lactate oxidase on the sensing electrode (Figure 4) [89]. Among the three configurations tested, the CNT/Ag-AgCl two-electrode system showed the best performance, with a lactate detection range of 47.6 µM–1.52 mM, a detection limit of 2.5 µM, and a sensitivity of 0.358 µA mM^−1^, while the three-electrode CNT/CNT/Ag-AgCl system showed a detection limit of 6.0 µM and sensitivity of 0.262 µA mM^−1^. The glove sensor also showed good stability under mechanical deformation and successfully measured lactate in real human sweat collected after different exercise conditions, supporting its potential for wearable sweat metabolite monitoring.

Uric acid has also been explored as a popular sweat biomarker because of its potential relevance to gout, cardiovascular disease, type 2 diabetes, and renal health [90,91,92]. Zeng et al. developed a CoWO_4_@CNT-based hydrogel-modified electrochemical sensor for simultaneous detection of uric acid and tyrosine (a sweat-detectable amino acid marker of amino acid metabolism) in real sweat [93]. The sensor showed analytical detection of uric acid over 1–1000 µM with an LOD of 0.14 µM and tyrosine over 5–1000 µM with an LOD of 4.2 µM, along with good selectivity against common sweat interferents. Integration with a polydopamine–polyacrylamide (PDA-PAM) hydrogel improved sweat collection, and real sweat testing further supported the sensor’s potential for health monitoring and real-time sweat analysis. In another example, Bi et al. developed a CNT-induced enzyme-polymerized wearable sweat biosensor for multifunctional monitoring of uric acid, glucose, and pH [94]. The device achieved 0.1 µM LODs for both glucose and uric acid over a linear range of 0–100 µM, with sensitivities of 2.07 nA µM^−1^ and 22.17 nA µM^−1^, respectively, and the pH sensor operated over a pH range of 4.0–8.0 with 51.1 mV pH^−1^ sensitivity. The integrated wearable platform was further evaluated through on-body sweat collection and analysis involving six human volunteers, demonstrating its feasibility for real-time sweat analysis.

Electrolyte monitoring is another important application area for CNT-based sweat sensors since Na^+^, K^+^, and Cl^−^ are relevant to hydration and electrolyte loss, while sweat chloride is the most clinically established sweat analyte because of its role in cystic fibrosis testing via the quantitative pilocarpine iontophoresis test (QPIT) [95,96,97,98]. However, electrolyte monitoring in sweat is commonly performed using CNT-based solid-contact ion-selective sensors rather than classical biosensors, and is therefore beyond the scope of this review [98].

Hormone monitoring in sweat is less explored but increasingly important. Cortisol is a major example. It is a stress biomarker and plays a crucial role in a variety of physiological processes, including metabolism in multiple organs, and blood pressure and blood sugar level regulation [99,100,101]. Su et al. developed a wearable electrochemical cortisol aptasensor based on Ni-Co metal–organic framework (MOF) nanosheets decorated on a CNT/polyurethane film [99]. The sensor showed a linear range of 0.1–100 ng mL^−1^ and a detection limit of 0.032 ng mL^−1^ in buffer, and successful pilot monitoring of cortisol in volunteer sweat, demonstrating the platform’s potential for quantitative stress monitoring and management.

Overall, sweat is one of the most advanced biofluids for CNT-enabled wearable sensing. The field has progressed from benchtop electrode demonstrations to flexible devices capable of real-sweat analysis, on-body measurements, and wireless monitoring. Several commercial sweat-monitoring products are now available for hydration and athletic-performance tracking [102,103,104]. However, CNT-based sweat biosensors remain largely at the research-prototype stage. Routine clinical use is still limited by sweat-rate variability, evaporation, skin contamination, biofouling, calibration across individuals, and uncertain correlations between many sweat biomarkers and blood-based clinical standards [17,87,105]. Therefore, CNT-based sweat biosensors are currently viewed as promising platforms for non-invasive and wearable monitoring, with further clinical validation needed before broader use in healthcare.

### 3.2. Saliva

Saliva is an information-rich non-invasive biofluid containing clinically relevant metabolites, enzymes, antibodies, cytokines, hormones, nucleic acids, exosomes, and pathogen-derived molecules [106,107,108]. It can provide information on both local oral conditions and systemic disease, including oral cancer, diabetes, infectious disease, renal disease, endocrine status, and neurodegenerative disorders [106,108]. Its painless and repeatable collection makes it attractive for point-of-care testing, longitudinal monitoring, and home-based diagnostics; however, saliva is also a complex analytical matrix. Whole saliva contains proteins, mucins, electrolytes, oral microorganisms, epithelial cells, and food debris and its composition also varies with flow rate, collection method, gland of origin, oral hygiene, diet, time of day and medication use, which affect viscosity, promote nonspecific adsorption, and complicate biomarker quantification [108,109,110,111]. Therefore, salivary CNT biosensors require not only sensitive transducers but also careful surface functionalization, antifouling strategies, and standardized collection protocols.

**Figure 5:**
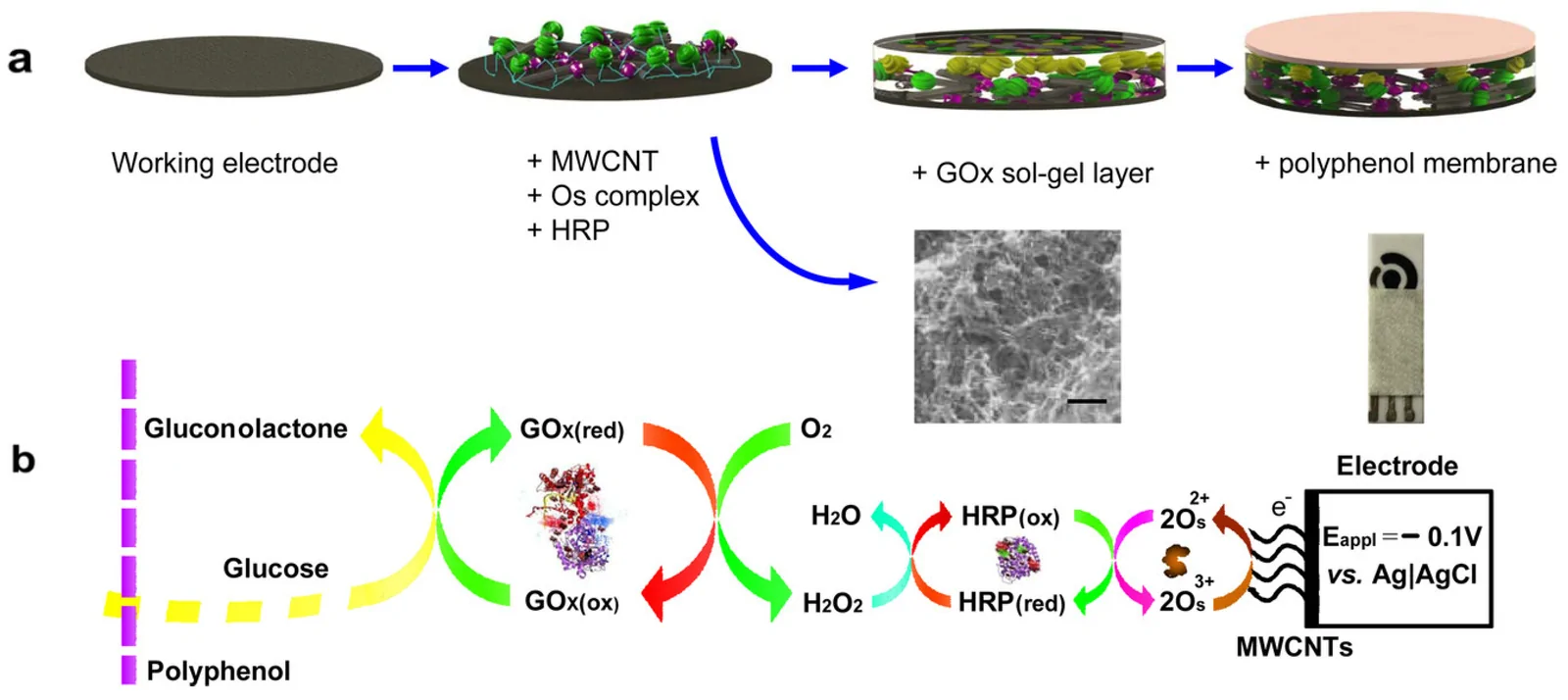
A bienzyme CNT/Os complex thin-film electrochemical biosensor for continuous glucose monitoring in human saliva. (**a**) Fabrication process and reagent layer constituents of the working electrode. (**b**) Response mechanism of the sensor. Symbols: GOx_(ox)_, GOx_(red)_, HRP_(ox)_ and HRP_(red)_ are the oxidized and reduced forms of the mentioned enzymes. Scale bar is 200 nm. (Reproduced with permission from ref. [114]).

Glucose has been one of the most studied salivary analytes because of its potential use in non-invasive diabetes monitoring, though its concentration in saliva (0.23–0.38 mM in healthy people and 0.55–1.77 mM in diabetics) is considerably lower compared to blood glucose (3.9–5.5 mM in healthy adults) [85,86,111,112]. Several CNT-based electrochemical platforms have demonstrated glucose detection within salivary concentration ranges. For instance, Liu et al. developed a salivary glucose biosensor based on Nafion-carbon nanotube (Nf-CNT) nanocomposite and glucose oxidase coated on a screen-printed electrode (SPE) [113]. The sensor showed high sensitivity of 99.13 µA mM^−1^ cm^−2^ in the range of 20–700 µM glucose. The electrodes were also tested in artificial saliva, highlighting their potential in the non-invasive diagnosis of diabetes. Du et al. reported an on-chip disposable salivary glucose biosensor where the working electrode was functionalized through a layer-by-layer assembly of SWCNTs and multilayer films of chitosan, gold nanoparticles and glucose oxidase [111]. The sensor showed a linear range of 0.5–20 mg dL^−1^ in buffer with an LOD of 0.41 mg dL^−1^ and detected 1.1–45 mg dL^−1^ glucose in saliva, supporting its potential as an adjunct non-invasive glucose monitoring tool. In another study by Liu et al., an electrochemical salivary glucose biosensor was described based on a bienzyme system with CNTs incorporated into an osmium (Os) complex thin film (Figure 5) [114]. The sensor achieved a linear range of 0.05–1.5 mM, detection limit of 0.003 mM, good selectivity against interferents and correlation with blood glucose, supporting its potential for continuous glucose detection in human saliva.

Beyond glucose, CNT-based biosensors have been applied to stress and inflammatory biomarkers in saliva, for example, cortisol, which is a common indicator of stress [115]. In this context, Tlili et al. developed a chemiresistive SWCNT immunosensor for rapid detection of salivary cortisol [115]. The CNTs were functionalized with a cortisol analog and anti-cortisol antibody, and free cortisol in buffer or artificial saliva displaced the antibody, producing a measurable resistance/conductance change. The sensor achieved an ultralow LOD of 1 pg mL^−1^ in artificial saliva and showed good selectivity against structurally similar steroids, supporting its potential for point-of-use stress/cortisol monitoring in saliva.

CNT biosensors have also been explored for disease-relevant salivary proteins. Rabbani et al. reported a CNT-FET immunosensor for salivary lysozyme, a potential new biomarker associated with Alzheimer’s disease [116,117]. The device used a Nafion/CNT nanocomposite on a polyethylene terephthalate (PET) substrate with anti-lysozyme antibodies immobilized on the CNT surface, achieving a linear detection range of 0.05–100 µg mL^−1^, a detection limit of 0.05 µg mL^−1^ and satisfactory recovery in spiked human saliva samples, suggesting significant potential applications in clinical diagnostics [116].

CNT-based immunosensors have also been reported for salivary cancer biomarkers. Viswanathan et al. developed a disposable electrochemical immunosensor for carcinoembryonic antigen (CEA) in saliva and serum using a polyethyleneimine-wrapped MWCNT screen-printed electrode and ferrocene-loaded liposome labels [118]. The reported calibration range was 5 × 10^−12^ to 5 × 10^−7^ g mL^−1^, with a detection limit of 1 × 10^−12^ g mL^−1^ in buffer. The immunosensor was also validated and tested in human saliva samples from both oral cancer patients and healthy volunteers. However, broader clinical validation would be required before diagnostic use.

Saliva is also important for infectious disease testing because it can contain viral antigens, antibodies, and nucleic acids. For example, Zamzami et al. developed a CNT FET-based biosensor for SARS-CoV-2 spike S1 antigen using 1-Pyrenebutanoic acid succinimidyl ester (PBASE) to immobilize anti-S1 antibodies on the CNT channel [61]. The device detected S1 antigen over a concentration range of 0.1 fg mL^−1^ to 5.0 pg mL^−1^ in 10 mM ammonium acetate buffer, with a detection limit of 4.12 fg mL^−1^. Its applicability to saliva testing was further demonstrated using healthy human saliva samples spiked with known amounts of S1 antigen, with a reported sensor readout time of 2–3 min. Although the platform was not evaluated using clinical saliva samples from infected patients, it shows great potential for rapid saliva-based detection of SARS-CoV-2 S1 antigen.

Overall, saliva is a highly attractive clinical biofluid for CNT-based biosensors because it provides access to metabolic, inflammatory, oncologic, neurodegenerative, and infectious disease markers through non-invasive collection. However, clinical translation remains limited by matrix complexity and fouling, sample viscosity, collection-site variability, and inconsistent biomarker correlations with blood or disease state. Future salivary CNT biosensors will require standardized collection protocols, real human saliva validation, antifouling surface chemistries, and comparison with established clinical assays before they can be reliably used for diagnostic decision-making. Thus, while salivary CNT biosensors have shown strong analytical sensitivity across several biomarker classes, most of them still remain at the proof-of-concept or early validation stage.

### 3.3. Tears

Tears provide a minimally invasive route for accessing biomarkers related to ocular surface health and, to some extent, systemic physiology [119,120]. However, tears are a technically demanding matrix for biosensor development. They are available in small quantities, biomarker concentrations are often low, and tear composition can change due to reflex tearing, evaporation, and ocular irritation [17,121]. In addition, ocular devices must satisfy strict requirements for transparency, oxygen permeability, flexibility, comfort, and biocompatibility [17,122]. These constraints make CNT-based tear biosensing more challenging than other fluids.

Glucose has been an attractive target for tear biosensors because tear glucose may reflect systemic glucose fluctuations and support non-invasive diabetic monitoring [17,123,124]. However, glucose concentrations in tears are much lower than in blood, typically 0.15–0.70 mM in healthy individuals and 0.8–1.5 mM in diabetic individuals, compared with approximately 3.9–5.5 mM in blood of healthy adults [85,86]. Given that CNTs can largely improve sensor performance as well as enable flexible and miniaturized device formats, CNT-based glucose sensors can be especially useful in this context [125,126]. However, CNT-based tear glucose sensing remains largely unexplored. This represents a promising gap, as properly prepared CNT films can combine flexibility, optical transparency, and electrical conductivity to be well suited for future wearable or contact-lens-based tear sensors [126].

**Figure 6:**
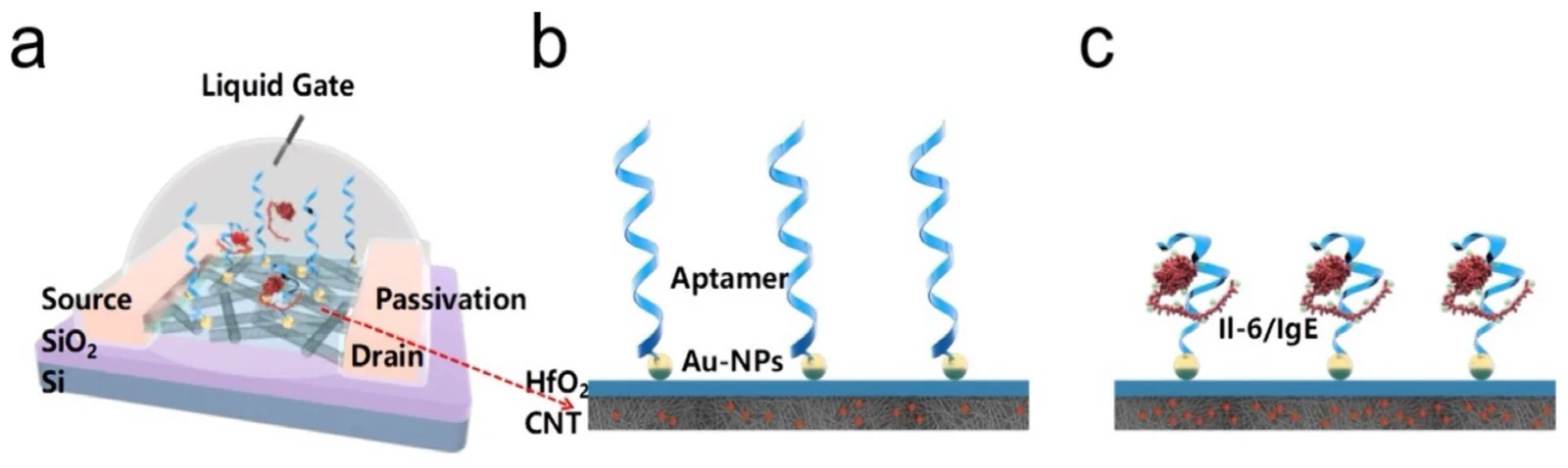
Schematic of the working principle of the aptamer-functionalized CNT-FET biosensor for IL-6 and IgE detection (**a**–**c**). (Adapted with permission from ref. [129]. Copyright 2026 American Chemical Society).

Tears also contain a variety of inflammatory markers such as matrix metalloproteinases (MMP) (e.g., MMP-9), cytokines (e.g., IL-1β, IL-6, IL-8) and immunoglobulin E (IgE) [127,128]. CNT-based biosensors have been developed for tear-based detection of these ophthalmic biomarkers. In a recent study by Zhu et al., the authors reported an aptamer-functionalized CNT-FET biosensor targeting IL-6 and IgE (Figure 6) [129]. To improve reproducibility, they introduced an ICP-O_2_ plasma and O_3_ interface-cleaning strategy, which reduced fabrication-related contamination, improved CNT-FET electrical properties, and increased the number of available aptamer-binding sites. The optimized biosensor detected trace IL-6/IgE proteins in complex tear samples with an ultralow LOD of 1.37 aM and showed good agreement with enzyme-linked immunosorbent assay (ELISA), supporting its potential for on-site ophthalmic disease diagnostics.

Tear-based point-of-care tests are already used clinically for dry-eye assessment, including tear osmolarity testing and MMP-9 inflammatory testing [130,131,132]. However, CNT-based tear biosensors remain comparatively underdeveloped, but they offer strong potential for future ocular and systemic health monitoring. Their ability to provide sensitive electrical transduction, support bioreceptor immobilization, and enable flexible device designs make them promising for next-generation tear sensing technologies.

### 3.4. Urine

Urine is one of the most established non-invasive biofluids in clinical diagnostics and is routinely used for the screening, diagnosis, and monitoring of kidney disease, urinary tract infections (UTI), pregnancy, metabolic disorders, and other health conditions. It is easy to collect, available in larger volumes than sweat, saliva, or tears, and contains clinically useful analytes including metabolites, proteins, hormones, and electrolytes [6,133]. Because point-of-care urine testing is already performed using dipsticks, strips, and cartridge-based assays, CNT-based urinary biosensors are mainly being developed to improve the sensitivity, selectivity, speed, and portability of diagnostic formats [6,134,135].

**Figure 7:**
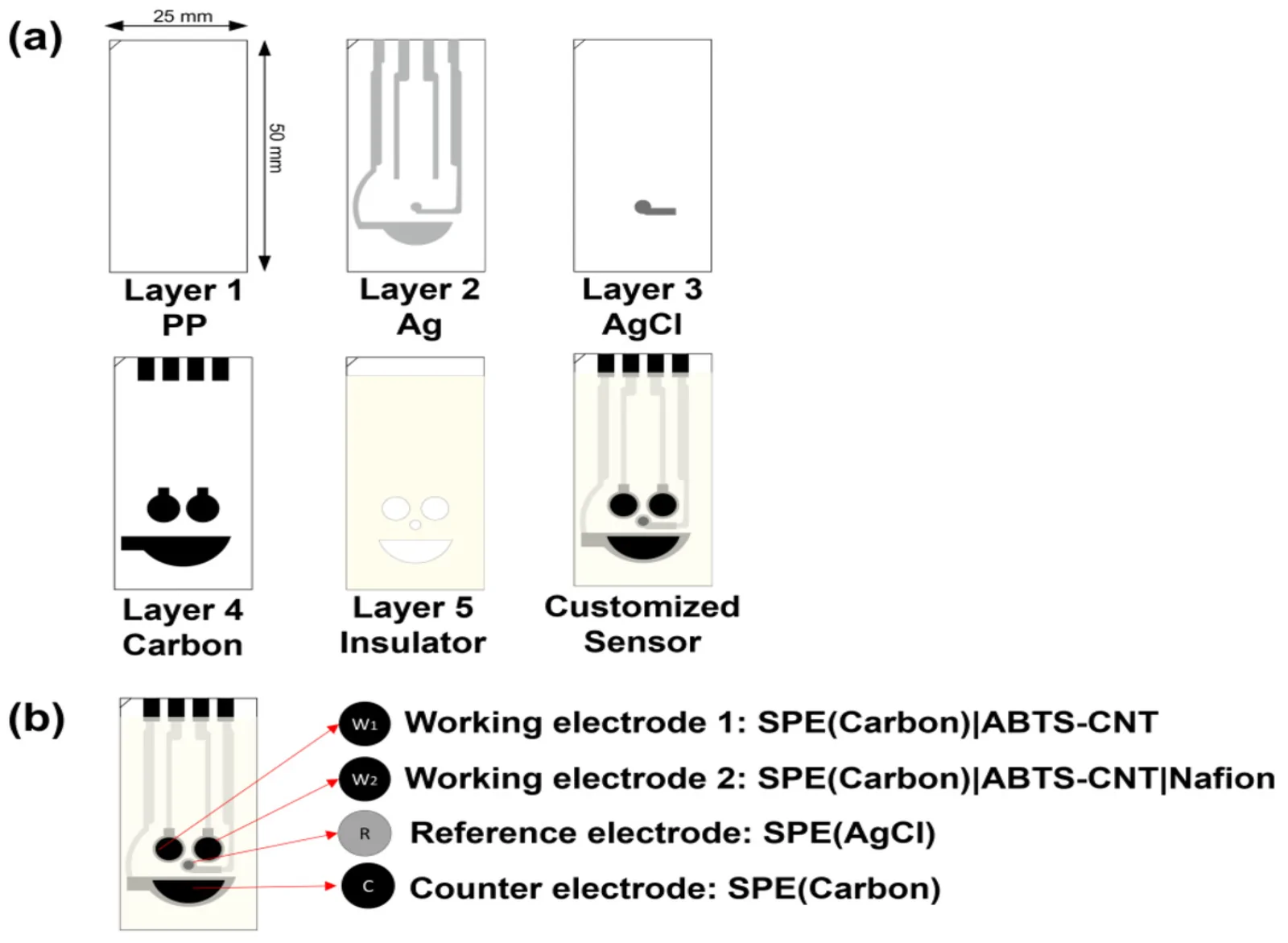
ABTS-modified electrochemical sensing platform for point-of-care UACR assessment. (**a**) Layer-by-layer configuration of the customized CNT-based electrode. (**b**) Schematic design of the dual-working-electrode electrochemical sensor for UACR detection. (Reproduced from ref. [136]).

A major focus of CNT-based urinary biosensing is kidney function assessment, and the urine albumin (Alb)-to-creatinine (Crn) ratio (UACR) is a sensitive and early indicator of chronic kidney disease (CKD) and cardiorenal syndrome [136,137,138]. Lee et al. developed a portable, wireless, and low-cost CNT/2,2′-azino-bis(3-ethylbenzothiazoline-6-sulphonic acid) (ABTS)-modified electrochemical sensing platform for point-of-care UACR assessment (Figure 7) [136,139]. The Alb sensor showed a linear range of 0.08–19.2 mg dL^−1^ with an LOD of 0.02 mg dL^−1^, while the Crn sensor showed a linear range of 1.67–58.62 mg dL^−1^ with an LOD of 0.62 mg dL^−1^. In spot urine samples from 30 diabetic patients, the UACR results showed excellent agreement with a biochemical analyzer. With auto-detection operation, simple storage, high accuracy, and a low manufacturing cost of <$0.50, the platform shows immense promise for practical point-of-care urinalysis and early CKD/cardiorenal risk assessment.

Metabolic analytes are another important class of urinary targets. Uric acid is clinically relevant to gout, hyperuricemia, renal dysfunction, and purine metabolism disorders [57,140]. Fukuda and co-workers developed an enzymatic CNT-based amperometric biosensor for uric acid detection in urine and serum [140]. The electrode used uricase immobilized in a CNT/carboxymethylcellulose (CMC) film, giving a linear range of 0.02–2.7 mM and an LOD of 2.8 µM. The biosensor was also successfully applied to human urine, making it an excellent example of CNT-enabled urinary uric acid biosensing. More recently, Zhang et al. developed a multiplexed and in situ self-calibrated biosensing system (MSSCBS) for metabolic health analysis in human urine, integrating eight electrochemical channels for the simultaneous detection of glucose, ketone bodies, bilirubin, ascorbic acid, uric acid, nitrite, calcium ions, and pH [141]. CNT-containing conductive interfaces were incorporated into six of the eight channels: a polyvinylpyrrolidone–Prussian blue–chitosan–CNT architecture for glucose; polyvinylpyrrolidone–Toluidine blue–chitosan–CNT architectures for ketone-body and bilirubin detection; and a hexadecyl trimethyl ammonium bromide–graphene oxide–chitosan–CNT architecture for enzyme-free detection of ascorbic acid, uric acid, and nitrite. The latter provided a reported uric acid limit of detection of 2.16 µM in phosphate-buffered saline. To address ascorbic acid-driven cross-interference in untreated urine, a dynamic normalized least mean squares algorithm used the ascorbic acid signal as an in situ reference for self-calibration of the uric acid, glucose, ketone-body, bilirubin, and nitrite channels. The array was subsequently evaluated in untreated human urine from healthy participants and patients with diabetes and kidney disease. This multiplexed CNT-integrated platform illustrates the growing development of comprehensive, self-calibrating urinary biosensing systems that extend beyond single-analyte detection toward broader metabolic profiling.

Urinary infection detection has also been explored using CNT-based sensors. Thulasinathan et al. developed an electrophoretically deposited MWCNT-based reagent-free electrochemical aptasensor for point-of-care detection of *Pseudomonas aeruginosa* in urine samples [142]. *Pseudomonas aeruginosa* is a Gram-negative opportunistic pathogen that commonly causes severe infections in immunocompromised patients and individuals with chronic diseases, and its antibiotic-resistance mechanisms often make these infections difficult to manage, making easy and rapid detection of the pathogen necessary [142,143]. The sensor showed a wide linear range of 10^1^–10^7^ CFU mL^−1^ with an LOD of 1 CFU mL^−1^, and enabled highly specific direct detection of the pathogen in undiluted human urine [142]. Proof-of-concept testing on disposable absorbent materials, including baby diapers and sanitary pads, further highlighted its potential for rapid UTI screening and point-of-care urinary pathogen detection.

Human chorionic gonadotropin (hCG), the hormone commonly used as a pregnancy biomarker, has also been detected using CNT-based urinary immunosensors [144]. In a study by Teixeira et al., a CNT screen-printed electrode was modified with APTES to enable near-oriented immobilization of anti-hCG antibodies [145]. The resulting immunosensor showed a linear response to hCG from 0.01 × 10^−9^ g cm^−3^ to 100 × 10^−9^ g cm^−3^, with only a modest decrease in sensitivity from buffer to synthetic urine and real urine (buffer > synthetic urine > real urine), negligible nonspecific binding, and successful hCG detection in urine from a pregnant volunteer. Overall, the biosensor’s analytical performance supports its potential use as a point-of-care diagnostic platform.

Urine also presents matrix-specific analytical challenges. Its composition varies with hydration status, diet, renal concentrating ability, collection timing, and disease state [6,133,146]. These changes affect analyte dilution, pH, ionic strength, and conductivity, all of which can influence sensor response. Urine also contains electroactive interferents, suspended cells, proteins, salts, and microbial products, which may affect sensor response and reproducibility; therefore, selective membranes, antifouling coatings, dilution protocols, or calibration strategies are necessary [146,147]. In Lee et al.’s UACR system, for example, urine samples were diluted fivefold in phosphate buffer to stabilize pH and conductivity, and Nafion was used to reduce interference in creatinine detection [136].

Overall, urine is an extremely promising biofluid for CNT-based biosensor translation. Future progress will depend on including broader panel of markers, larger clinical studies, robust comparison with established urinalysis methods, improved antifouling and anti-interference strategies, and standardized calibration across diverse patient populations.

**Table 2:** Comparative analytical performance and validation of representative CNT-based biosensors for non-invasive health monitoring and disease-related biofluid analysis.

Biofluid	Analyte/Target	CNT Material	Sensing Method	Linear Range/LOD	Validation Matrix	Ref.
**Sweat**	Glucose	MWCNT/PEDOT:PSS/Fe_3_O_4_	Electrochemical biosensor	1–400 µM; LOD 0.38 µM. Artificial sweat: 10–200 µM; LOD 0.31 µM	Human sweat	[84]
**Sweat**	Glucose	PGA-CNT/GOx	Enzymatic electrochemical biosensor	20–300 µM; LOD 5.18 µM	Artificial sweat	[88]
**Sweat**	Lactate	CNT/Ag–AgCl with lactate oxidase	Amperometric biosensor	47.6 µM–1.52 mM; LOD 2.5 µM	Human sweat	[89]
**Sweat**	Uric acid/Tyrosine	CoWO_4_@CNT hydrogel	Electrochemical sensor	UA: 1–1000 µM; LOD 0.14 µM. Tyr: 5–1000 µM; LOD 4.2 µM	Human sweat	[93]
**Sweat**	Glucose/Uric acid/pH	CNT-induced enzyme-polymerized platform	Multifunctional electrochemical biosensor	Glucose and UA: 0–100 µM; LOD 0.1 µM each. pH: 4.0–8.0	Human sweat	[94]
**Sweat**	Cortisol	Ni-Co MOF/CNT-polyurethane	Electrochemical aptasensor	0.1–100 ng mL^−1^; LOD 0.032 ng mL^−1^	Human sweat	[99]
**Saliva**	Glucose	Nafion-CNT/GOx	Enzymatic electrochemical biosensor	20–700 µM	Artificial saliva	[113]
**Saliva**	Glucose	SWCNT/chitosan/AuNP/GOx	Enzymatic electrochemical biosensor	0.5–20 mg dL^−1^; LOD 0.41 mg dL^−1^	Human Saliva	[111]
**Saliva**	Glucose	CNT/Os-complex bienzyme film	Bienzymatic electrochemical biosensor	0.05–1.5 mM; LOD 0.003 mM	Human saliva	[114]
**Saliva**	Cortisol	Cortisol-functionalized SWCNTs	Competitive chemiresistive immunosensor	LOD 1 pg mL^−1^ in artificial saliva	Artificial saliva	[115]
**Saliva**	Lysozyme	Nafion/CNT with anti-lysozyme antibodies	CNT-FET immunosensor	0.05–100 µg m L^−1^; LOD 0.05 µg mL^−1^	Spiked human saliva	[116]
**Saliva**	Carcinoembryonic antigen	PEI-wrapped MWCNT electrode	Electrochemical immunosensor	5 × 10^−12^–5 × 10^−7^ g mL^−1^; LOD 1 × 10^−12^ g mL^−1^	Saliva from oral-cancer patients and healthy volunteers	[118]
**Saliva**	SARS-CoV-2 S1 antigen	PBASE/anti-S1-functionalized CNT	CNT-FET immunosensor	0.1 fg m L^−1^–5.0 pg mL^−1^; LOD 4.12 fg mL^−1^	Spiked healthy human saliva	[61]
**Tears**	IL-6/IgE	Aptamer-functionalized CNT	CNT-FET aptasensor	LOD 1.37 aM in complex tear samples	Human tear	[129]
**Urine**	Albumin/Creatinine	CNT/ABTS dual-electrode platform	Electrochemical sensing	Albumin: 0.08–19.2 mg dL^−1^; LOD 0.02 mg dL^−1^. Creatinine: 1.67–58.62 mg dL^−1^; LOD 0.62 mg dL^−1^	Spot urine from 30 diabetic patients	[136,139]
**Urine**	Uric acid	CNT/CMC film with uricase	Enzymatic amperometric biosensor	0.02–2.7 mM; LOD 2.8 µM	Human urine	[140]
**Urine**	Glucose, ketone bodies, bilirubin, ascorbic acid, uric acid, nitrite, Ca^2+^, and pH	CNT-containing multichannel interfaces	Multiplexed electrochemical sensing with in-situ self-calibration	UA LOD 2.16 µM	Untreated urine from healthy participants and patients with diabetes or kidney disease	[141]
**Urine**	*Pseudomonas aeruginosa*	Aptamer-functionalized MWCNT electrode	Electrochemical aptasensor	10^1^–10^7^ CFU mL^−1^; LOD 1 CFU mL^−1^	Undiluted human urine	[142]
**Urine**	Human chorionic gonadotropin	APTES-modified CNT electrode with anti-hCG antibodies	Electrochemical immunosensor	0.01–100 ng mL^−1^	Synthetic urine and urine from a pregnant volunteer	[145]

Note: Unless otherwise specified, the reported LODs were determined in buffer. Abbreviations: ABTS, 2,2′-azino-bis(3-ethylbenzothiazoline-6-sulfonic acid); APTES, (3-aminopropyl)triethoxysilane; AuNP, gold nanoparticle; CFU, colony-forming unit; CMC, carboxymethylcellulose; CNT, carbon nanotube; ELISA, enzyme-linked immunosorbent assay; FET, field-effect transistor; GOx, glucose oxidase; hCG, human chorionic gonadotropin; IgE, immunoglobulin E; IL-6, interleukin-6; LOD, limit of detection; MOF, metal–organic framework; MWCNT, multi-walled carbon nanotube; PBASE, 1-pyrenebutanoic acid succinimidyl ester; PBS, phosphate-buffered saline; PEDOT:PSS, poly(3,4-ethylenedioxythiophene):polystyrene sulfonate; PEI, polyethyleneimine; PGA, polyglycolic acid; SWCNT, single-walled carbon nanotube; Tyr, tyrosine; UA, uric acid.

## 4. Applications of CNT-Based Non-Invasive Biofluid Biosensors Beyond Clinical Diagnostics

Beyond disease diagnosis and clinical monitoring, CNT-based biofluid biosensors are also being explored for forensic identification and toxicology, exposure assessment, and public-safety applications. In these settings, the goal is to detect exogenous compounds, identify biological evidence, or monitor chemical exposure using accessible matrices such as sweat, saliva, urine, or deposited body-fluid traces. The following sections discuss application areas such as drug-of-abuse detection, forensic body-fluid identification, and occupational or environmental exposure assessment.

### 4.1. Drug-of-Abuse Detection

Non-invasive biofluids such as sweat, saliva, and urine are attractive matrices for drug-of-abuse testing, providing information on recent or cumulative exposure without blood collection. Urine is the most widely used biological matrix for drug testing, while sweat and saliva are increasingly explored for rapid, field-deployable, and non-invasive screening [148]. However, it is important to note that in this area, most CNT-based drug sensors remain at the laboratory or early-prototype stage. Table 3 summarizes representative CNT-based sensing platforms for drug-of-abuse testing, forensic body-fluid identification, and occupational or environmental exposure assessment, including their targets, CNT materials, sensing methods, analytical performance, and validation matrices.

In a recent study on opioid detection in artificial sweat matrix, Shao et al. developed gold nanoparticle-decorated, semiconductor-enriched SWCNT FET (Au-SWCNT FET) sensor arrays functionalized with opioid-specific antibodies [11]. In the norfentanyl sensor, norfentanyl antibodies were immobilized on Au-SWCNT FETs, enabling sensitive detection of norfentanyl, the primary fentanyl metabolite. Using an optimized protocol involving 10 min sample incubation, a washing step to reduce matrix interference, and FET measurement in diluted PBS gating solution, the sensor achieved a detection limit of 34 pg mL^−1^ (146 pM) and a working range of 0.385–753 pg mL^−1^ in artificial sweat. The platform was further expanded into a three-antibody functionalized sensor array targeting norfentanyl, morphine, and 6-monoacetylmorphine. Because of antibody cross-reactivity with structurally related compounds, the array could also detect the corresponding parent opioids, fentanyl, codeine, and heroin. Integration with automated and portable FET measurement formats further highlighted its potential for rapid, non-invasive opioid screening, although direct validation in authentic human sweat remains an important next step. Earlier work from the same group reported a norfentanyl antibody-functionalized SWCNT-FET biosensor that achieved femtogram-per-milliliter sensitivity in PBS calibration samples and synthetic urine, demonstrating the potential of CNT-FET platforms for urinary fentanyl exposure screening [149].

For most other drugs, CNT electrochemical platforms remain largely confined to seized samples or plasma rather than non-invasive biofluids [150,151].

Overall, CNT biosensor architectures show strong promise for ultrasensitive forensic drug sensing but direct validation in authentic sweat, saliva, or urine remains limited. Future progress will depend on testing these platforms in real biofluid samples, improving antifouling and anti-interference performance, and integrating validated sensors into portable formats suitable for field-based screening.

### 4.2. Forensic Body Fluid Identification

Forensic body-fluid identification is another emerging application area for CNT-based biosensors, where the aim is to determine the biological origin of trace evidence at crime scenes.

For instance, Dutta et al. developed multiplexed, paper-based chemiresistive nanobiosensor arrays using SWCNT-antibody bio-ink, functionalizing SWCNTs with antibodies against fluid-specific protein biomarkers including α-amylase (for saliva), osteopontin (for urine), and dermcidin (for sweat). Antigen binding modulated chemiresistor conductance, enabling label-free, multiplexed identification of analytes, with limits of detection in low micro- to nanomolar scales. The sensors were also validated with real human samples under practical case-like conditions [152,153].

More recently, Govender et al. reported a CNT-based electrochemical sensor for forensic saliva identification by targeting *Streptococcus salivarius*, a saliva-associated bacterial species [154]. The sensor used GH12 antimicrobial peptide immobilized on carboxyl-functionalized CNTs. Using differential pulse voltammetry, the sensor achieved a detection limit of 0.15 McF and a 40-min response time, and successfully identified saliva within mixed body fluid samples, though a decline in performance was noted with desiccated or improperly stored saliva samples.

These studies illustrate how CNT-based biosensors can enable complementary approaches to forensic body-fluid identification: multiplexed protein-biomarker detection for broad body-fluid screening and bacterial-marker detection for saliva-specific confirmation in mixed samples. Moving forward, researchers will need to address challenges that are central to forensic translation, including performance with aged and degraded samples, variable substrates, reagent stability, and wireless readout. Addressing these issues will be pivotal in transitioning CNT-based body-fluid identification from proof-of-concept studies toward practical crime-scene or laboratory forensic workflows.

### 4.3. Occupational and Environmental Exposure Assessment

CNT-based biosensors also have considerable potential for occupational and environmental exposure assessment. This is important because organophosphate (OP) pesticides and nerve agents can inhibit cholinesterase activity, leading to serious neurotoxic effects; so, rapid biomonitoring is needed to identify exposure early and guide timely intervention to prevent permanent neurological damage [155,156]. In this context, non-invasive biofluids such as saliva and urine are particularly important. However, it must be noted that CNT-based exposure-monitoring biosensors in authentic non-invasive biofluids remain less explored.

A notable example was reported by Wang et el. who developed a highly sensitive CNT-based electrochemical sensor for measuring salivary cholinesterase activity as an exposure biomarker for OP pesticides and nerve agents [12]. Using CNT-modified screen-printed carbon electrode in a microflow-injection system, the platform detected thiocholine, the electroactive product of acetylthiocholine hydrolysis, with the CNTs enhancing its oxidation at low potential for sensitive amperometric detection. Calibrated with purified acetylcholinesterase (AChE) in buffer, the sensor achieved a linear range of 5 pM to 0.5 nM and a detection limit of approximately 2 pM. It was further validated in native rat saliva, remaining sensitive even at 40-fold dilution, and tracked real-time enzyme inhibition following OP paraoxon exposure.

The same research direction was extended by Du et al. using CNT-modified screen-printed electrode coupled to flow-injection amperometric detection for biomonitoring organophosphorus exposure through cholinesterase reactivation [157]. Rat saliva was used as an in vitro biological matrix, and the system measured both inhibited and reactivated cholinesterase activity after treatment with pralidoxime iodide (antidote to OP poisoning). Paraoxon was used as a model organophosphate compound, and the optimized system achieved 92–95% cholinesterase reactivation over a broad inhibition range of 5–94%. The authors reported that the sensor could detect paraoxon inhibition efficiency in saliva samples and proposed the platform for point-of-care, non-invasive biomonitoring of exposure to organophosphate pesticides and chemical nerve agents.

There is clear potential of CNT-based biosensors in the area of exposure assessment. Future work should therefore focus on testing and translating these platforms in authentic human biofluids, improving antifouling and anti-interference performance, validating results against established reference methods, and integrating the sensors into portable formats suitable for field-based use.

**Table 3:** Comparative analytical performance and validation of representative CNT-based non-invasive biofluid sensing platforms beyond clinical diagnostics.

Application/Biofluid	Analyte/Target	CNT Material	Sensing Method	Linear Range/LOD	Validation Matrix	Ref.
**Drug-of-abuse detection/Sweat**	Norfentanyl, morphine, and 6-monoacetylmorphine	Au-decorated, semiconductor-enriched SWCNTs with opioid-specific antibodies	CNT-FET sensor array	Norfentanyl: 0.385–753 pg mL^−1^; LOD 34 pg mL^−1^ in artificial sweat	Artificial sweat	[11]
**Drug-of-abuse detection/Urine**	Norfentanyl	Antibody-functionalized SWCNTs	CNT-FET biosensor	LOD 2.0–3.7 fg mL^−1^	PBS and synthetic urine	[149]
**Forensic body-fluid identification/Saliva, urine, and sweat**	α-Amylase, osteopontin, and dermcidin	Antibody-functionalized SWCNT bio-ink on paper	Multiplexed chemiresistive biosensor array	Target-dependent; LODs in the low micromolar to nanomolar range	Human body-fluid samples under case-like conditions	[152,153]
**Forensic saliva identification/Saliva**	*Streptococcus salivarius*	Carboxyl-functionalized CNTs with GH12 antimicrobial peptide	Differential-pulse-voltammetric electrochemical sensor	LOD 0.15 McF	Mixed body-fluid samples	[154]
**Exposure assessment/Saliva**	Cholinesterase activity	CNT-modified screen-printed carbon electrode	Microflow-injection amperometric enzyme-activity sensor	5 pM–0.5 nM; LOD 2 pM	Purified AChE in buffer; native rat saliva	[12]
**Exposure assessment/Saliva**	Inhibited and reactivated cholinesterase activity	CNT-modified screen-printed electrode	Flow-injection amperometric enzyme-activity sensor	92–95% reactivation over a 5–94% inhibition range	Rat saliva	[157]

Note: Unless otherwise specified, the reported LODs were determined in buffer. Abbreviations: AChE, acetylcholinesterase; Au, gold; CNT, carbon nanotube; FET, field-effect transistor; LOD, limit of detection; McF, McFarland unit; PBS, phosphate-buffered saline; SWCNT, single-walled carbon nanotube.

## 5. Challenges, Strategies and Future Outlook

Despite strong progress, CNT-based biosensors for non-invasive biofluid analysis still face several barriers that must be addressed to facilitate their real-world translation and widespread use. The following sections outline key challenges along with future directions for moving these CNT-based biosensors from laboratory demonstrations toward practical applications.

### 5.1. Material Reproducibility and Standardization

Over the past several years, there has been remarkable progress in the development of CNT-based biosensors; however, reproducibility remains a major translational challenge [28,30]. This is largely due to the intrinsic CNT heterogeneity, including variation in chirality, metallic-to-semiconducting ratio, tube length, bundling, defect density, and residual catalyst impurities [28,29,158]. These factors influence conductivity, optical emission, electrochemical behavior, baseline signal, and analyte response leading to device-to-device and batch-to-batch variability [22,24,25,26,159]. Variability can also arise during sensor fabrication, especially during CNT dispersion, film formation, and surface functionalization. Differences in linker chemistry, receptor density, orientation, and nonspecific adsorption can alter bioreceptor accessibility and sensor response [22,28,30,159]. Therefore, reproducible CNT biosensor fabrication requires not only consistent CNT materials but also standardized processing and biofunctionalization workflows.

Purification techniques, such as polymer-assisted sorting, density gradient centrifugation, and column chromatography have improved CNT yield, but these methods are often time-consuming and increase cost and complexity [28,160,161]. Future work should therefore prioritize scalable and low-cost purification strategies, for example, chemically modified cotton-wool-based separation, to produce more uniform CNT fractions for reproducible biosensor fabrication [28,162]. Transparent reporting standards are also essential for cross-study comparison. Key parameters such as CNT source, purification method, diameter/chirality distribution, metallic/semiconducting fraction, defect density, dispersion protocol, ink concentration, and substrate type should be routinely provided [163]. Standardized CNT inks, reference devices, and consensus testing protocols will further support reliable performance evaluation [164]. Reproducibility should be demonstrated not only through repeated measurements on a single device but also across independently fabricated devices, fabrication batches, operators, and CNT material lots. Together, these efforts will be critical for translating CNT-based biosensors from laboratory demonstrations to scalable and clinically useful technologies.

### 5.2. Biofouling and Matrix Effects

Real biofluids are chemically complex and can interfere with CNT biosensor performance. Sweat, saliva, tears, and urine contain proteins, salts, lipids, mucins, metabolites, cells, and microbial components that can adsorb onto CNT surfaces or recognition layers, causing biofouling, biocorona formation, baseline drift, reduced sensitivity, and loss of specificity [31]. These effects are especially important for non-invasive biofluid monitoring because sensors may be used for repeated sampling or continuous on-body measurements, where surface fouling, receptor degradation, and impaired analyte transport can accumulate over time [15]. As a result, performance demonstrated in clean buffer or short single-use assays may not accurately predict long-term behavior in real sweat, saliva, tears, or urine.

Antifouling strategies such as polyethylene glycol (PEG) coatings, zwitterionic polymers, hydrogels, protective membranes, and hydrophilic interfaces can reduce nonspecific adsorption while still allowing target analytes to reach the sensing interface [28,165]. As a recent example from a related carbon-based field-effect transistor platform, Wang et al. developed an aptamer-functionalized graphene field-effect transistor incorporating a biomolecule-permeable PEG layer to reduce nonspecific adsorption and increase the effective Debye screening length. The optimized device detected tumor necrosis factor-α and interleukin-6 in undiluted physiological media (undiluted artificial sweat and undiluted lavage fluid), with reported limits of detection of 0.13 and 0.20 pM, respectively, highlighting the value of engineered polymer interfaces for sensing in complex biofluids [166]. Concepts from long-term implantable biosensors also highlight the importance of designing interfaces that resist protein adsorption, cell adhesion, and other biological deposits while preserving sensor permeability and signal transduction [165]. To ensure widespread adoption of CNT-based sensors, future studies should move beyond buffer-only testing and validate the biosensors under biofluid-specific conditions, including repeated exposure, storage, variable pH and ionic strength, and relevant interferents. Long-term reliability will depend on the durability of recognition layers, antifouling interfaces, and calibration strategies tailored to each target biofluid.

### 5.3. Sampling and Physiological Interpretation

Non-invasive biofluid sensing requires careful control of sample collection and calibration because measured analyte concentrations depend not only on physiological status but also on fluid volume, secretion rate, collection method and duration, time of day, diet, hydration, medication use, physical activity, and individual physiology. The sampling procedure itself can substantially alter the measured composition [1]. For example, stimulated and unstimulated saliva may differ in flow rate and analyte concentration, while reflex tears may be diluted relative to basal tears [16,167]. Therefore, results obtained using different collection procedures cannot always be directly compared. Kessler et al. tested whether meal timing affects salivary biomarkers by giving participants controlled diets and collecting saliva every 4 h over a 24 h period. They found that several biomarkers changed across the day, showing that saliva composition is strongly time-dependent [168]. These findings support the need to standardize sampling protocols when developing or validating biosensors.

Inter-individual variability represents an additional challenge [169]. A sensor evaluated using samples from one individual or a small number of volunteers may demonstrate feasibility but cannot establish that the same calibration or diagnostic threshold will apply across a broader population. Future validation should therefore include samples from multiple individuals, repeated measurements from the same individuals on different days, and relevant physiological and demographic variation. Such studies are needed to distinguish true biomarker-related changes from normal within-person and between-person variability.

Calibration procedures must also reflect the intended sample matrix. Calibration curves prepared in buffer may not accurately represent sensor responses in authentic biofluids because of differences in pH, ionic strength, viscosity, fouling, and analyte recovery. Depending on the application, reliable quantification may require matrix-matched calibration, standard addition, internal reference channels, correction for temperature and pH, normalization for secretion rate or urine dilution, or comparison with an individual baseline. CNT-based biosensors should therefore be validated in authentic biofluids and compared with established reference methods. Because the relationship between biofluid analyte concentrations and systemic physiology is analyte-specific and often incompletely defined, standardized sampling, appropriate calibration, and realistic validation are essential for reliable interpretation [1].

### 5.4. Biocompatibility, Safety, and Regulation

CNT safety depends on tube length, diameter, aspect ratio, aggregation state, surface chemistry, catalyst content, dose, and exposure route [170,171,172,173,174]. In most non-invasive biosensors, CNTs are immobilized within electrodes, inks, or membranes, which may limit direct exposure, but application-specific evaluation is still needed for skin-contact, intraoral, ocular, wearable, and disposable devices. Potential concerns include particle release, irritation during prolonged contact, and environmental accumulation [175,176,177].

These risks may be reduced through CNT purification, biocompatible functionalization, encapsulation, protective membranes, and contact-limiting designs [22,178,179]. Biodegradation of CNTs by microbes (e.g., naphthalene-degrading bacteria) and enzymes (e.g., horseradish peroxidase) has also been reported, highlighting a potential route to reduce their biological persistence and environmental burden [180]. Regulatory translation will ultimately require clear documentation of CNT source, purity, immobilization stability, release risk, shelf life, and device-to-device variability [28,181].

### 5.5. Scalable Manufacturing and System Integration

Moving CNT biosensors beyond laboratory prototypes will require scalable and reproducible manufacturing. Printing methods such as screen printing, inkjet printing, and roll-to-roll processing are promising for flexible, wearable, paper-based, and disposable sensors; however, they require careful quality control of CNT ink stability, film uniformity, receptor loading, shelf life, and batch-to-batch response [28,182,183,184,185].

Practical CNT biosensors must also be designed as complete sensing systems rather than isolated sensing interfaces. Depending on the target biofluid and application, this may require integration with sample collection, fluid handling, signal readout, calibration, power supply, wireless communication, and data processing. For example, for sweat sensors, microfluidic architecture can help guide small sweat volumes to the sensing area, reduce evaporation, and improve sensing efficiency during continuous monitoring [19,20,186,187].

Multiplexed CNT sensor arrays are also important for clinical, forensic, and exposure monitoring applications, where single-analyte measurements may be insufficient. However, these systems require careful control of cross-reactivity, signal interference, calibration drift, and device-to-device variability [28,188].

### 5.6. Future Outlook

The future of CNT-based non-invasive biofluid biosensing will increasingly depend on the development of portable, wireless, and connected sensing systems. Integration with compact electronics, low-power readout circuits, Bluetooth/Wi-Fi/5G communication, smartphone interfaces, and cloud-based data platforms can enable real-time monitoring, remote data access, and more practical use in field settings [28].

Internet of Things (IoT)-enabled CNT biosensors will be especially valuable for applications that require continuous or repeated measurements, such as sweat-based health monitoring and occupational exposure screening. By linking CNT sensor outputs with wearable devices, mobile phones, or remote healthcare platforms, these systems can support faster decision-making, personalized tracking, and field-deployable analysis outside centralized laboratories [28,189,190].

Artificial intelligence (AI) and machine learning (ML) may further improve the interpretation of CNT biosensor data [28,191,192]. These tools can help reduce noise, correct signal drift, recognize multianalyte patterns, and classify complex biofluid signatures. However, AI-based models will only be reliable if they are trained using authentic biofluids, diverse users, reference-method comparisons, and real-world testing conditions; therefore, AI should complement, not replace, rigorous analytical validation.

Overall, the real-world impact of CNT-based biofluid biosensors will depend on combining connected system design with reproducible CNT materials, antifouling interfaces, scalable manufacturing, multiplexed sensing, physiological calibration, safety evaluation, and robust validation in real samples.

## 6. Conclusions

CNT-based biosensors have demonstrated considerable potential for non-invasive biofluid analysis because their electronic, electrochemical, optical, and surface properties support sensitive detection in compact and flexible formats. Across sweat, saliva, tears, and urine, CNT-enabled platforms have been developed for diverse biomarkers using field-effect transistor, chemiresistive, electrochemical, and optical transduction mechanisms. Emerging applications in drug-of-abuse detection, forensic body-fluid identification, and occupational or environmental exposure assessment further illustrate the versatility of these technologies. However, the studies reviewed range from proof-of-concept testing in buffer or artificial biofluids to measurements in authentic human samples and small on-body or clinical studies. Analytical sensitivity alone should therefore not be considered evidence of readiness for practical use.

Taken together, no single CNT sensing architecture has yet demonstrated the combined robustness, reproducibility, and validation required for routine or commercial non-invasive testing. The available literature suggests that electrochemical platforms have been evaluated more frequently in integrated devices and authentic biofluids, whereas CNT-FET, chemiresistive, and optical systems remain at more variable and often earlier stages of validation. This should not be interpreted as a definitive ranking, because the reported studies differ substantially in their target analytes, sample matrices, device formats, intended applications, and evaluation criteria. The most appropriate sensing approach will ultimately depend on the biofluid, biomarker, sampling strategy, and intended use.

Future progress will require attention to the complete analytical workflow rather than improvements in transducer sensitivity alone. Standardized sample collection, effective control of biofouling and matrix interference, consideration of inter-individual physiological variability, reliable calibration, and reproducibility across independently fabricated devices and CNT batches will be essential. Long-term stability, scalable manufacturing, validation in larger and more diverse populations, and comparison with established reference methods will also be needed. Continued integration with microfluidics, multiplexed sensing, portable and wireless electronics, and AI-assisted data analysis may further improve device operation and interpretation. Addressing these challenges could enable CNT-based biosensors to progress from promising research prototypes toward reliable platforms for non-invasive diagnostics, health monitoring, and field-based analysis.

## Data Availability

No new data were created or analyzed in this study. Data sharing is not applicable to this article.
